# Re‐Visiting the Content Validity of the Manchester Clinical Supervision Scale (MCSS‐26)

**DOI:** 10.1111/inm.70128

**Published:** 2025-09-04

**Authors:** Niels Buus, Hosu Ryu, Roshani Prematunga, Wendy Ducat, Marcus Gardner, Henrik Gonge, Bridget Hamilton, Rebecca J. Jarden, Priya Martin, Sarah Osiurak, David A. Snowdon

**Affiliations:** ^1^ Department of Public Health Aarhus University Aarhus Denmark; ^2^ School of Nursing and Midwifery Monash University Melbourne Victoria Australia; ^3^ School of Nursing and Midwifery La Trobe University Melbourne Victoria Australia; ^4^ Centre for Mental Health Nursing University of Melbourne Melbourne Victoria Australia; ^5^ Mental Health Learning West Moreton Hospital and Health Service Ipswich Queensland Australia; ^6^ Bendigo Health Bendigo Victoria Australia; ^7^ Gonge Consultancy Aarhus Denmark; ^8^ Department of Nursing University of Melbourne Melbourne Victoria Australia; ^9^ Austin Health Heidelberg Victoria Australia; ^10^ Occupational Therapy University of Southern Queensland Toowoomba Queensland Australia; ^11^ Physiotherapy Department, Eastern Health Box Hill Melbourne Australia; ^12^ School of Allied Health, Human Services and Sport La Trobe University Melbourne Victoria Australia; ^13^ Academic Unit, Peninsula Health Melbourne Victoria Australia

**Keywords:** organisation and administration, preceptorship, questionnaire, questionnaire design, validation study

## Abstract

Clinical supervision is widely regarded as an important part of both pre‐graduate and post‐registration education and training of healthcare professionals. To ensure comprehensive implementation of effective supervision practices, it is crucial that supervisors, healthcare organisations and researchers have valid and reliable instruments to measure these practices. The Manchester Clinical Supervision Scale (MCSS) is the most widely used instrument for measuring supervision effectiveness in nursing and allied health. According to the developers of MCSS, it is based on Proctor's three functions of supervision as being normative, formative and restorative. The purpose of this paper was to report a test of the content validity of MCSS‐26, which is the latest version. Methods included: 1. A qualitative text analysis of MCSS‐26's syntax and wording. 2. A Content Validity Index with an expert panel rating the relevance of MCSS‐26 items for measuring effectiveness of supervision and their clarity. 3. A linguistic reordering of items and a tabulation of panel classifications of MCSS‐26 items according to Proctor's three functions. Findings revealed heterogeneity in MCSS‐26's wording and an uneven flow with negative/general questions being frontloaded. The CVI identified 46% of items (*n* = 12/26) as relevant for directly or indirectly measuring effectiveness of clinical supervision. The expert panel was not able to consistently link items to Proctor's functions. The results have important implications for how to interpret MCSS‐26 ratings of effectiveness of clinical supervision and can be used to consider psychometric studies examining the potential for an abbreviated version of MCSS‐26 with a single focus on effectiveness.

## 1 Introduction

Clinical supervision is considered a central part of health and social care professionals' continual learning and development (Rothwell et al. 2021). Clinical supervision has been recognised as an important practice for professional growth for mental health nurses since the 1980s (Masamha et al. 2022). Despite longstanding interests and wide endorsement by professional bodies (Australian College of Mental Health Nurses et al. 2024), there is varying evidence regarding clinical supervision's effect on patient care and safety (Watkins 2020). With such endorsement, there is a growing demand for quality evaluation of its effectiveness. The Manchester Clinical Supervision Scale (MCSS) is the leading measurement instrument for evaluating clinical supervision (Winstanley and White 2011) in nursing (Edgar et al. 2024) and allied health (Snowdon et al. 2016). It has been used widely in mental health nursing research and evaluation contexts (Berry and Robertson 2019; Gonge and Buus 2015, 2016; Hamilton et al. 2023; Ryu et al. 2024; White and Winstanley 2010).

MCSS was originally a 36‐item questionnaire designed to measure respondents' perceived effectiveness of clinical supervision (Winstanley 2000); it was later reduced to 26 items based on an analysis of its psychometric performance (Winstanley and White 2011). Given its dominance in the field, we wish to ensure MCSS‐26's continued relevance by testing MCSS‐26's content validity by explicating and examining the supervision construct that it measures.

## 2 Background

Understanding the historical context of the Manchester Clinical Supervision Scale (MCSS) ensures its validity and relevance by revealing the original assumptions, cultural norms and theoretical foundations that shaped its development. Prior to the MCSS, there was no standardised measurement to adequately assess its effectiveness. The design of MCSS was one of the major outcomes of a large evaluation of clinical supervision and mentorship in England and Scotland between 1995 and 1997, ‘It is good to talk’ (Butterworth et al. 1997). The aims of the evaluation included an exploration of evaluation tools that could be used for measuring the impact of clinical supervision, and to report on supervision‐related activities at the 23 participating clinical sites (Butterworth et al. 1997; White et al. 1998) involving a total of 586 nurses and health visitors from different clinical specialties. Participating sites had to provide clinical supervision defined as: (1) A written contract between supervisors and supervisees, (2) No less than 45 min of supervision every 4 weeks, and (3) Supervision and mentorship actively address the normative, formative and restorative needs of the supervisees (Butterworth et al. 1997). Data collection included site questionnaires, repeated standardised and in‐house surveys, and individual interviews. Thematic analysis of the interviews with participants indicated that while they had very little experience with clinical supervision before it was offered to them as part of the evaluation, they knew of similar activities. Respondents were largely in favour of clinical supervision but would also emphasise practical difficulties of making it happen in a busy work environment (White et al. 1998). The key issue here is that MCSS was developed and validated in organisational contexts where clinical supervision practices were not yet rigorously implemented, and what counted as clinical supervision was very broadly defined. In terms of content validity, this means that MCSS might not capture all the important dimensions of fully implemented supervision, or over‐ or under emphasise certain dimensions. For example, it may measure the perceived value of supervision rather than the perceived effectiveness of supervision.

Qualitative data from the evaluation were collated and turned into 59 statements about clinical supervision. The ‘MCSS‐59’ was administered to 467 nurses and was reduced to 45 items following an explorative factor analysis with varimax rotation. ‘MCSS‐45’ was administered to an additional 560 respondents and factor analysis was repeated, first using the new dataset and later using the merged dataset. This identified 36 items and factor structure with seven subscales (Winstanley 2000). Reliability analyses were made using the merged dataset. Cronbach's alpha for the total 36‐item scale was 0.86, with sub‐scales ranging from 0.6975 to 0.9078. Using 45 sets of paired questionnaires, intraclass correlation coefficients were calculated as ranging between 0.777 and 0.93. The authors attempted to evaluate the face validity of MCSS‐36 by inspecting the qualitative data of the six participants who reported extreme scores: 3 with lowest total scores and 3 with highest total scores (Winstanley 2000). This approach to analysing face validity was in effect confirmatory and did not challenge the instrument's design.

The evaluation had a tendency to favour Proctor's (1987) supervision framework, stipulating that supervisor and supervisee take up different roles and responsibilities, as they collaboratively engage with a variety of tasks. However, the conceptual links between MCSS and Proctor's framework have never been demonstrated or tested. According to Proctor (1987), these joint tasks can emphasise normative (maintaining professional and ethical standards), formative (addressing education and skill‐building) and restorative (addressing the supervisee's emotional and psychological well‐being) functions. Proctor did not envision that these tasks were mutually exclusive; rather, they were integrated and potentially conflicting (Proctor 1987). Originally, the MCSS sub‐scales were linked to these three functions, with two out of seven subscales being linked to more than one supervision task (Winstanley 2000), which aligned with Proctor's ideas about the multiple joint tasks for supervisors and supervisees. Later, each subscale was linked exclusively to only one of Proctor's functions. What complicates the issue is not only the overlapping functions, but also the variety of practices that are grouped under the term ‘clinical supervision’ (Ryu et al. 2024; Zonneveld et al. 2025). This is a fundamental problem when considering construct validity, as it makes it harder to ensure that a questionnaire's items cover all relevant aspects of the construct (Haynes et al. 1995).

Furthermore, there are significant overlaps between the qualitative findings from the evaluation (White et al. 1998) and the titles of MCSS sub‐scales, which may indicate a data‐driven development of MCSS focused on structure, process and outcome, rather than a theory‐driven approach based on Proctor's framework. For instance, interviewees were concerned about time, 'the difficulty in making time available for such [supervision] sessions, in competition with time for direct patient care, was the issue most frequently reported’ (White et al. 1998, 190). These concerns were translated into negative MCSS items regarding ‘finding time’.

Finally, Winstanley and White (2011) conducted a Rasch item response analysis and reduced the number of items to 26 and the number of sub‐scales to six. This approach was a continuation of the data‐driven approach described above. Despite claims that MCSS‐26 is better aligned with Proctor's tasks than MCSS‐36, the removal of 10 items improved MCSS's psychometric properties. However, it did not fundamentally deal with the relationship to Proctor's concepts. The aim of the current paper is therefore to conduct a test of the English language MCSS‐26's construct validity. We anticipate that an analysis can assist in improved interpretations of MCSS‐26 studies, and that findings can provide insights into the conduct of future supervision questionnaire design.

## 3 Methods

This article reports an evaluation of the content validity of MCSS‐26. The evaluation includes a linguistic analysis of the wording and syntax of MCSS‐26's items, an expert assessment of the degree to which MCSS‐26 items are relevant, clear and representative of supervision effectiveness (a content validity index, CVI), and, finally, a linguistic re‐ordering of items and a tabulation of panel classifications of MCSS‐26 items according to Proctor's three functions.

### 3.1 MCSS‐26

The MCSS‐26 is for individuals currently receiving clinical supervision and it consists of two sections. Section A includes 26 items about the effectiveness of the respondent's clinical supervision. A five‐point Likert scale is used, and there is no option for responding ‘not relevant’. Section B includes 20 items concerning the respondent's demographics and work experiences, the respondent's supervisor and the respondent's clinical supervision sessions. Section A can be calculated as a total score, according to the six subscales or according to Proctor's three functions. Section B items can be used to contextualise the Section A scores.

### 3.2 Analysis of Syntax

The analysis of syntax explored how words and phrases were arranged in the English version of MCSS‐26, and explored the interface between syntax and semantics (Carnie 2021). These analyses were conducted by the first author.

### 3.3 Expert Panel

In October 2024, we assembled a panel with 10 international (8 Australian and 2 Danish), interdisciplinary clinical supervision experts and researchers: 2 clinical psychologists, 4 nurses, 1 occupational therapist, 2 physiotherapists and 1 podiatrist. Eight had a PhD degree. In total, the panel members had published 71 peer‐reviewed articles and 7 book chapters, reports or commentaries on clinical supervision.

### 3.4 Item Level CVI: Data and Statistical Analysis

A post hoc Content Validity Index assesses the adequacy of a questionnaire and can help identify potential weaknesses that may have gone unnoticed during initial development (Lynn 1986). Specifically, we test if MCSS items are aligned with its explicitly stated focus on effectiveness, identify items that do not reach defined thresholds, and suggest revisions, deletions or substitutions (Polit and Beck 2006).

Data were gathered from the 10 members of the panel (Lynn 1986), each of whom independently evaluated the ‘relevance for measuring (directly or indirectly) the effectiveness of clinical supervision (its process or outcome)’ and clarity of 26 items from the MCSS‐26 using a 4‐point Likert scale:

1 = Not relevant/Not clear.

2 = Somewhat relevant/Needs major clarification.

3 = Mostly relevant/Needs minor clarification.

4 = Very relevant/Very clear.

Descriptive statistics, including the mean, standard deviation, median and interquartile range (IQR), were calculated for both relevance and clarity ratings using SPSS (IBM SPSS Statistics for Windows, Version 28.0). Given the non‐normal distribution of the data, non‐parametric tests were used, including the Friedman test and Kendall's W, to examine the consistency and agreement among raters. The Friedman test was used to compare the mean ranks for relevance and clarity ratings across raters. Kendall's W test was used to assess the level of agreement among raters. A *p*‐value of less than 0.05 was considered statistically significant for all tests.

To further assess inter‐rater reliability, Fleiss' Kappa was calculated. Fleiss' Kappa measures agreement beyond chance; the standard error (SE) and 95% confidence intervals (CI) were also calculated. Additionally, Cronbach's alpha was used to assess the internal consistency of the ratings, with values above 0.70 considered acceptable.

The Content Validity Index (CVI) was calculated based on the binary variables created from the relevance and clarity ratings. The binary variables were as follows:

- Relevance: Ratings of 1 and 2 were coded as 0 (not relevant, somewhat relevant); ratings of 3 and 4 were coded as 1 (mostly relevant, very relevant).
- Clarity: Ratings of 1 and 2 were coded as 0 (not clear, needs major clarification); ratings of 3 and 4 were coded as 1 (needs minor clarification, very clear).

CVI scores of 0.80 or higher were considered acceptable for both relevance and clarity ratings (Lynn 1986).

### 3.5 Qualitative Analysis: Reordering of Items and Tabulation According to Proctor

We identified the information structure of the items by identifying the ‘topic’ and ‘comment’ and used this to group the items linguistically rather than according to subscales. This linguistic reordering was then evaluated in relation to the CVI ratings (see Table 3). Then we tabulated how the panel members classified items according to Proctor's three domains in response to the question: ‘Which supervision function(s) do you intuitively believe the item belongs to (normative, formative, restorative, or something fourth)?’

## 4 Findings

### 4.1 Syntax and Item‐Order

All MSCC‐26 items are *declarative* (they make a statement directly or indirectly about supervision effectiveness) sentences, but the grammar of the items in MCSS‐26 is not homogeneous. All items are in the *active* voice, except for Item #10 ‘Work problems can be tackled constructively during CS sessions’, which is passive. All items are *simple*, except for Item #26 ‘I think receiving clinical supervision improves the quality of care I give’, which is a complex sentence (a sentence that contains one independent clause and at least one dependent clause). Also, there is a compound sentence with two independent clauses, Item #3 ‘CS sessions are not necessary/don't solve anything’.

The above‐mentioned use of the passive voice and the ‘I think’ hedges softens the force of the statement. In addition, four items include the modal verb ‘can’, which also hedges the force of a statement by expressing uncertainty. Hedging introduces a level of ambiguity in the question that may prompt respondents to interpret it in various ways (Oppenheim 1992).

Most items follow the basic subject‐verb‐object (SVO) order, which is intuitive for English speakers, as it reflects a straightforward progression of events that reduces ambiguity. See, for example, item #23 ‘CS sessions [S] motivate [V] staff [O]’. Most items are more complicated than a basic SVO structure, such as item #25 ‘My supervisor offers me guidance with patient/client care’, which has a subject‐verb‐indirect object‐direct object‐prepositional phrase structure. Simple, active SVO sentences are more readable, and the presence of complex, passive and compound sentences increases the cognitive load on the reader/responder (Oppenheim 1992).

Considering the syntax‐semantics interface, MCSS‐26 is heterogeneous with a mix of positive and negative items and of personal and general items. ‘Negative’ items make negative statements regarding supervision, and scores are reversed before analysis. Negative items are more complex and therefore harder to process for interpreters/respondents (Oppenheim 1992). MCSS‐26 includes 16 (62%) personal items and 10 (38%) general items, with a higher proportion of personal items in the second half (77%). Personal items are marked using ‘I’, ‘me’ and ‘my’, sometimes in combination. Item #13, ‘I can discuss sensitive issues encountered during my clinical casework with my supervisor’, includes three indicators of the item being personal. Conversely, the general statements are markedly more factual, with, for example, two instances of the ‘it is’ structure (#2 and #16), which adds to create neutral, impersonal statements.

MCSS‐26 includes 17 (65%) positive statements and 9 (35%) negative statements. The negative statements are primarily placed at the beginning of the questionnaire, with 7 of the first 8 items being negative (see Table 1). The high proportion of negative general questions early in the survey could create a pessimistic or critical tone and create a pronounced compounding negative effect on the reader/respondent's cognitive load in the first one‐third of the questionnaire, which could create biases or disengagement (Schleef 2013). However, as the item order moves respondents from negativity (general concerns) to positivity (personal experiences) it could potentially keep respondents motivated throughout the questionnaire (Schwarz et al. 1991).

**TABLE 1:** Composition of negative/positive and personal/general items in MCSS‐26 and CVI ratings for relevance and clarity.

		Value	Abstraction	Relevance CVI	Clarity CVI
1	Other work pressures interfere with CS sessions	Negative	General	0.5	0.8
2	It is difficult to find the time for CS sessions	Negative	General	0.3	0.9
3	CS sessions are not necessary/don't solve anything	Negative	General	0.7	0.7
4	Time spent on CS takes me away from my real work in the clinical area	Negative	Personal	0.6	0.7
5	Fitting CS sessions in can lead to more pressure at work	Negative	General	0.2	0.7
6	I find supervision sessions time‐consuming	Negative	Personal	0.3	0.9
7	My supervisor gives me support and encouragement	Positive	Personal	0.9	1.0
8	CS sessions are intrusive	Negative	General	0.3	0.5
9	Supervision gives me time to ‘reflect’	Positive	Personal	0.8	0.9
10	Work problems can be tackled constructively during CS sessions	Positive	General	1.0	1.0
11	CS sessions facilitate reflective practice	Positive	General	1.0	1.0
12	My supervisor offers an ‘unbiased’ opinion	Positive	Personal	0.5	0.8
13	I can discuss sensitive issues encountered during my clinical casework with my supervisor	Positive	Personal	0.9	1.0
14	My CS sessions are an important part of my work routine	Positive	Personal	0.9	1.0
15	I learn from my supervisor's experiences	Positive	Personal	0.9	1.0
16	It is important to make time for CS sessions	Positive	General	0.6	1.0
17	My supervisor provides me with valuable advice	Positive	Personal	0.7	1.0
18	My supervisor is very open with me	Positive	Personal	0.7	1.0
19	Sessions with my supervisor widen my clinical knowledge base	Positive	Personal	0.9	1.0
20	Supervision is unnecessary for experienced/established staff	Negative	General	0.3	0.7
21	My supervisor acts in a superior manner during our sessions	Negative	Personal	0.5	1.0
22	Clinical supervision makes me a better practitioner	Positive	Personal	0.9	1.0
23	CS sessions motivate staff	Positive	General	0.5	0.9
24	I can widen my skill base during my CS sessions	Positive	Personal	0.9	1.0
25	My supervisor offers me guidance with patient/client care	Positive	Personal	0.9	0.9
26	I think receiving clinical supervision improves the quality of care I give	Positive	Personal	1.0	1.0

### 4.2 Content Validity Index (CVI)

CVI scores for item relevancy and clarity are listed in Table 1. The descriptive statistics for the relevance and clarity ratings are summarised in Table 2.

**TABLE 2:** Summary of CVI scores for relevance and clarity.

CVI type	*N*	Minimum	Median	IQR	Maximum	Mean	Std. Dev.
Relevance	26	0.2	0.7	0.5–0.9	1	0.6808	0.25771
Clarity	26	0.5	1.0	0.8–1.0	1	0.9000	0.13856

For relevance, the minimum score was 0.2, the median was 0.7 and the interquartile range (IQR) was between 0.5 and 0.9. The mean score was 0.68 (SD = 0.26), indicating moderate agreement among raters on the relevance of the items. Individual mean item scores ranged from 2.08 to 3.65, with an overall average of 2.98. The standard deviations for individual items varied from 0.452 to 0.983, suggesting varying levels of agreement among raters. Fourteen items did not meet the 0.80 threshold for relevance, but several items scored a perfect CVI of 1.00, indicating strong agreement among raters.

For clarity, the minimum score was 0.5, the median was 1.0 and the IQR ranged from 0.8 to 1.0. The overall mean score was 0.90 (SD = 0.14), indicating high agreement among raters that most items were clear. Individual mean item scores ranged from 2.46 to 3.88, with an overall average of 3.28. Standard deviations for clarity ranged from 0.196 to 1.029, again indicating variability in how raters evaluated the items. While most items had near‐perfect CVI scores, five items did not meet the 0.80 threshold for clarity.

### 4.3 Inter‐Rater Agreement

The Fleiss' Kappa coefficient indicated slight agreement among raters for both relevance (*κ* = 0.082, SE = 0.024, 95% CI [0.034, 0.129]) and clarity (*κ* = 0.012, SE = 0.022, 95% CI [−0.032, 0.056]). According to the Landis and Koch (1977) scale, Kappa values between 0.00 and 0.20 indicate slight agreement; values between 0.21 and 0.40 indicate fair agreement. These results suggest considerable variation in how raters interpreted the relevance and clarity of the items.

### 4.4 Kendall's W

Kendall's W coefficient was used to assess the level of agreement among raters. Kendall's W coefficient showed moderate agreement among raters for both relevance (*W* = 0.381, *p* < 0.001) and clarity (*W* = 0.290, *p* < 0.001).

### 4.5 Differences in Ratings Across Raters: Friedman Test

The Friedman test was conducted to determine if there were significant differences in ratings across raters. The Friedman test indicated significant differences in ratings across the raters for both relevance (χ^2^(9) = 89.11, *p* < 0.001) and clarity (χ^2^(9) = 67.97, *p* < 0.001). For relevance, Rater 9 had the highest mean rank (7.81), while Rater 3 had the lowest (2.44). For clarity, Rater 9 again had the highest mean rank (7.81), and Rater 3 had the lowest (2.88). These findings suggest that raters scored the items differently, pointing to inconsistencies in how they interpreted them.

### 4.6 Internal Consistency

To assess how consistently the raters evaluated the items, Cronbach's alpha was calculated. The relevance ratings showed strong internal consistency (*α* = 0.875), while the clarity ratings showed acceptable consistency (*α* = 0.716).

### 4.7 Linguistic Reordering of Items and the Links to Proctor's Model

Our linguistic reclassification included five groups of items: 1. *Supervisor attributes*, 2. *Supervisee actions*, 3. *Supervision practices*, 4. *Circumstances* and 5. *Value of supervision* (see Table 3). This reordering had some commonalities with MCSS‐26's six subscales, with the former three covering ‘Trust/rapport’, ‘Supervisor advice/support’ and ‘Reflection’, respectively, and the latter two covering ‘Finding time’, ‘Importance/value of CS’ and ‘Improved care/skills’, respectively.

**TABLE 3:** Discursive reorganisation classification of items and CVI scores.

	Value	General vs. personal	CVI relevance	CVI clarity	MCSS‐26 subscale
Supervisor attributes:					
7. My supervisor gives me support and encouragement	Positive	Personal	0.9	1.0	Trust/rapport
12. My supervisor offers an ‘unbiased’ opinion	Positive	Personal	0.5	0.8	Trust/rapport
17. My supervisor provides me with valuable advice	Positive	Personal	0.7	1.0	Supervisor advice/support
18. My supervisor is very open with me	Positive	Personal	0.7	1.0	Trust/rapport
21. My supervisor acts in a superior manner during our sessions	Negative	Personal	0.5	1.0	Trust/rapport
25. My supervisor offers me guidance with patient/client care	Positive	Personal	0.9	0.9	Supervisor advice/support
Supervisee actions:					
13. I can discuss sensitive issues encountered during my clinical casework with my supervisor	Positive	Personal	0.9	1.0	Trust/rapport
15. I learn from my supervisor's experiences	Positive	Personal	0.9	1.0	Supervisor advice/support
24. I can widen my skill base during my CS sessions	Positive	Personal	0.9	1.0	Supervisor advice/support
Supervision practices:					
9. Supervision gives me time to ‘reflect’	Positive	Personal	0.8	0.9	Reflection
10. Work problems can be tackled constructively during CS sessions	Positive	General	1.0	1.0	Reflection
11. CS sessions facilitate reflective practice	Positive	General	1.0	1.0	Reflection
19. Sessions with my supervisor widen my clinical knowledge base	Positive	Personal	0.9	1.0	Supervisor advice/support
Circumstances:					
1. Other work pressures interfere with CS sessions	Negative	General	0.5	0.8	Finding time
2. It is difficult to find the time for CS sessions	Negative	General	0.3	0.9	Finding time
4. Time spent on CS takes me away from my real work in the clinical area	Negative	Personal	0.6	0.7	Importance/value of CS
5. Fitting CS sessions in can lead to more pressure at work	Negative	General	0.2	0.7	Finding time
Value of supervision:					
3. CS sessions are not necessary/don't solve anything	Negative	General	0.7	0.7	Importance/value of CS
6. I find supervision sessions time‐consuming	Negative	Personal	0.3	0.9	Finding time
8. CS sessions are intrusive	Negative	General	0.3	0.5	Importance/value of CS
14. My CS sessions are an important part of my work routine	Positive	Personal	0.9	1.0	Improved care/skills
16. It is important to make time for CS sessions	Positive	General	0.6	1.0	Importance/value of CS
20. Supervision is unnecessary for experienced/established staff	Negative	General	0.3	0.7	Importance/value of CS
22. Clinical supervision makes me a better practitioner	Positive	Personal	0.9	1.0	Improved care/skills
23. CS sessions motivate staff	Positive	General	0.5	0.9	Improved care/skills
26. I think receiving clinical supervision improves the quality of care I give	Positive	Personal	1.0	1.0	Improved care/skills

*Supervisor attributes* included all items beginning with ‘My supervisor…’, which described supervisor actions and behaviour, which often also depicted the relationship between the supervisor and the supervisee. Five of these items are positive, and all are personal. The CVI indicated that six items were clear, but that four items were not relevant in terms of directly addressing supervision effectiveness.

*Supervisee actions* included all items describing actions that supervisees can do during supervision, ‘I…’. All three items were positive and personal, and the CVI indicated that they were all relevant and clear. *Supervision practices* included items with topics related to supervision activities, mostly concerning reflection. There are four positive items, with two being personal and two being general.

*Circumstances* included items concerning actions related to making supervision happen, mostly concerning finding time, and, as stated previously, were placed right at the beginning of the questionnaire. These four items were all negative, with three being general and one being personal. The CVI indicated that these items were not regarded as relevant, and two were not regarded as clear.

Value of supervision included nine items evaluating supervision, with four negative items and four positive items. All negative items and two of the positive items were rated as not relevant; three of the negative items were also rated as unclear.

In summary, the 14 items that were rated as not relevant in the CVI were not evenly distributed according to the discursive classification of items. All nine negative items were rated as not relevant, and these items were related to *Circumstances* and *Value of supervision*. Also, most *My supervisor* items were rated as not relevant. Conversely, *Supervisee actions* and *Supervision practices*, predominantly positive and personal, were rated as relevant.

Finally, we were not able to consistently classify items according to Proctor's three domains (see Table 4). Table 4 shows that there is little consensus on the function(s) of CS that each item addresses, except for two items relating to the formative domain.

**TABLE 4:** Reviewer ratings of item alignment with Proctor's functions of clinical supervision (*n* = 10).

MCSS‐26 item (Proctor's function assigned by tool developer)	Restorative only *n* (%)	Formative only *n* (%)	Normative only *n* (%)	Restorative and formative *n* (%)	Restorative and normative *n* (%)	Formative and normative *n* (%)	All three functions *n* (%)	None *n* (%)
Normative items:								
1. Other work pressures interfere with CS sessions	1 (10%)	0	2 (20%)	0	0	0	4 (40%)	3 (30%)
2. It is difficult to find the time for CS sessions	0	0	2 (20%)	0	1 (10%)	0	3 (30%)	4 (40%)
3. CS sessions are not necessary/don't solve anything	0	0	2 (20%)	0	0	1 (10%)	4 (40%)	3 (30%)
4. Time spent on CS takes me away from my real work in the clinical area	0	2 (20%)	1 (10%)	0	1 (10%)	0	1 (10%)	5 (50%)
5. Fitting CS sessions in can lead to more pressure at work	3 (30%)	0	3 (30%)	0	0	0	0	4 (40%)
6. I find supervision sessions time‐consuming	2 (20%)	1 (10%)	1 (10%)	0	0	0	2 (20%)	4 (40%)
8. CS sessions are intrusive	2 (20%)	1 (10%)	0	0	0	1 (10%)	3 (30%)	3 (30%)
16. It is important to make time for CS sessions	0	0	1 (10%)	0	0	1 (10%)	3 (30%)	5 (50%)
20. Supervision is unnecessary for experienced/established staff	0	2 (20%)	1 (10%)	0	0	0	2 (20%)	5 (50%)
Restorative items:								
7. My supervisor gives me support and encouragement	5 (50%)	0	0	3 (30%)	0	0	1 (10%)	1 (10%)
12. My supervisor offers an ‘unbiased’ opinion	1 (10%)	0	1 (10%)	2 (20%)	1 (10%)	1 (10%)	2 (20%)	2 (20%)
13. I can discuss sensitive issues encountered during my clinical casework with my supervisor	1 (10%)	2 (20%)	1 (10%)	2 (20%)	0	0	4 (40%)	0
15. I learn from my supervisor's experiences	0	4 (40%)	0	1 (10%)	0	1 (10%)	4 (40%)	0
17. My supervisor provides me with valuable advice	0	4 (40%)	1 (10%)	0	0	0	4 (40%)	1 (10%)
18. My supervisor is very open with me	0	1 (10%)	0	1 (10%)	1 (10%)	0	3 (30%)	4 (40%)
19. Sessions with my supervisor widen my clinical knowledge base	0	9 (90%)	0	0	0	1 (10%)	0	0
21. My supervisor acts in a superior manner during our sessions	2 (20%)	1 (10%)	0	0	0	0	4 (40%)	3 (30%)
24. I can widen my skill base during my CS sessions	0	9 (90%)	0	0	0	0	1 (10%)	0
25. My supervisor offers me guidance with patient/client care	0	7 (70%)	0	0	0	2 (20%)	0	1 (10%)
Formative items:								
9. Supervision gives me time to ‘reflect’	0	5 (50%)	0	2 (20%)	0	0	3 (30%)	0
10. Work problems can be tackled constructively during CS sessions	2 (20%)	1 (10%)	1 (10%)	1 (10%)	1 (10%)	1 (10%)	3 (30%)	0
11. CS sessions facilitate reflective practice	0	5 (50%)	0	2 (20%)	0	0	3 (30%)	0
14. My CS sessions are an important part of my work routine	0	0	2 (20%)	0	0	0	3 (30%)	5 (50%)
22. Clinical supervision makes me a better practitioner	0	3 (30%)	1 (10%)	0	0	3 (30%)	3 (30%)	0
23. CS sessions motivate staff	2 (20%)	1 (10%)	0	0	0	0	3 (30%)	4 (40%)
26. I think receiving clinical supervision improves the quality of care I give	0	5 (50%)	2 (20%)	0	0	1 (10%)	1 (10%)	1 (10%)

## 5 Discussion

Our analyses revealed heterogeneity in MCSS‐26's wording and an uneven flow with negative/general questions about *Circumstances* being frontloaded. Moreover, the CVI identified only 46% of items (12/26) as relevant for directly or indirectly measuring the effectiveness of clinical supervision, with these items primarily being related to *Supervisee actions* and *Supervision practices*. Finally, the expert panel was not able to consistently link items directly to Proctor's functions.

Considering MCSS‐26's item design, the use of hedging introduces a level of ambiguity, meaning that individuals might understand these questions differently. Differences in understanding could result in inconsistencies in the data and challenges in the analyses. Also, the mix of personal and general items can confuse the responder and potentially lead to inconsistent responses (Oppenheim 1992). Similar concerns were raised following a pilot for a trial of online learning versus blended learning of clinical supervisee skills with pre‐registration nursing students (McCutcheon et al. 2018). MCSS‐36 was administered to participants in the pilot, and their responses made the authors modify the scale for the trial. These modifications included: rewording all compounded items; removing superfluous markers of a personal item; and changing the tense of 16 items to present/future rather than present using ‘could’, ‘would’ and ‘should’ (McCutcheon et al. 2018). For instance, ‘CS should give me time to reflect’ rather than ‘CS gives me time to “reflect”’. While we recognise an intuitive urge to repair parts of MCSS's syntax and wording, the use of, for instance, different modal verbs alters items significantly, reduces comparability and creates new content validity issues.

Conducting post hoc CVIs can strengthen confidence in an instrument, but they can also introduce new problems, for instance, loss of cultural or historical comparability or unintended construct shifts (Lynn 1986). The CVI indicated that while raters demonstrated moderate internal consistency in their assessments, inter‐rater agreement was low, as indicated by Fleiss' Kappa. This suggests considerable variation in how different raters interpreted the relevance and clarity of the MCSS‐26 items. These findings highlight the need for further refinement or clarification of item wording to improve consistency (Polit and Beck 2006); however, the CVI should not be used to conduct any significant changes to MCSS‐26 without making use of other validation methods, preferably in collaboration with the original developers of the instrument.

A formal CVI was not part of the original design of the MCSS items and questionnaire, but we are aware of two previous MCSS CVIs conducted in relation to translations of MCSS that also identified content validity concerns. Hyrkäs, Appelqvist‐Schmidlechner, and Oksa (2003); Hyrkäs, Appelqvist‐Schmidlechner, and Paunonen‐Ilmonen (2003) translated the early, 45‐item version of MCSS into Finnish with a procedure that included a qualitative and quantitative CVI of MCSS‐36 involving 11 experts. This process led to the elimination of a subscale, ‘Finding time’, and at least 10 items that were deemed unacceptable in terms of clarity, concreteness, centrality and importance. The experts also found that ‘it was important to re‐phrase, re‐word, or develop and add new items’ (Hyrkäs, Appelqvist‐Schmidlechner, and Oksa 2003, 623) about the relationship between clinical supervision and the quality of care. Through this process, the Finnish MCSS was amended to 33 items. Khani et al. (2009) reported the translation of MCSS‐36 into Iranian, which included an evaluation (CVI) by five experts. The CVI indicated that while all items were deemed relevant, four items were not important, but Khani et al. (2009) did not state how they addressed items that were ‘not important’. While the development of MCSS‐26 undoubtedly solved some of the interpretation/equivalence issues, the differences between these two responses to sub‐threshold CVI scores are striking. Hyrkäs, Appelqvist‐Schmidlechner, and Oksa (2003) altered the questionnaire significantly to have a better fit with local practices, and Khani et al. (2009) seemed to ignore the identified problems. We suggest a more balanced approach that maintains a basic level of historical comparability while improving clarity, which includes factor analysis of the 12 items that were deemed relevant for measuring supervision effectiveness. This could confirm whether these items exhibit strong internal consistency and be used to update the instrument to ensure that items are proportionally distributed across the major dimensions of supervision effectiveness.

MCSS‐26 is designed to measure supervisees' perception of the effectiveness of clinical supervision, but our findings raise concerns about whether the development of the MCSS was appropriately guided by theory. The combination of items related to *Supervisor attributes*, *Supervisee actions*, *Supervision practices*, *Circumstances* and *Value of supervision* suggested that MCSS measures more variables than effectiveness. Our findings also highlighted that not all MCSS‐26 items are directly and consistently linked to Proctor's theory regarding three functions of supervision.

Our findings have important implications for how to interpret MCSS scores. Researchers and evaluators should be cautious when interpreting MCSS total scores, as not all items may be relevant to effectiveness of clinical supervision. However, 12 items did reach the threshold for relevance, suggesting that the total score is, at worst, somewhat reflective of the effectiveness of clinical supervision. Further, total scores have been shown to correlate with reduced levels of burnout, which would be an expected outcome of effective supervision (Martin et al. 2021). The subscale scores are more problematic; two of the six subscales, ‘finding time’ and ‘importance/value of clinical supervision’, did not have a single relevant item. These subscales may not be reflective of effective clinical supervision. Finally, items could not be consistently linked to Proctor's functions, and only a couple could be linked to a single function, suggesting that MCSS items cannot be summed to provide an indication of effectiveness for a single function.

## 6 Strengths and Limitations

The observed variation in how different raters interpreted the relevance and clarity of the MCSS‐26 items could be explained by unfamiliarity with the rating criteria or by raters having different understandings of supervision practices. The former could potentially have been addressed with more extensive rater training, and the latter points to the challenges of measuring different practices using a single instrument. However, it was a strength of the evaluation that the panel had specialist clinical supervision expertise, knew Proctor's conceptual framework, were familiar with the MCSS‐26 and that the instructions for panel members regarding the CVI relevance ratings were clear: to rate items in relation to ‘the effectiveness of clinical supervision’ (cf., Grant and Davis 1997). It should be noted that the CVI's focus on effectiveness meant that it did not evaluate its relevance for measuring other dimensions of clinical supervision, for instance, MCSS‐26 as a measure of value or satisfaction with supervision. The interdisciplinary composition of the group meant that panel members brought different emphases into their evaluations as they had different practical understandings of clinical supervision practices. For instance, physiotherapists were more accustomed to clinical supervision as part of co‐practice between supervisor and supervisee than panel members with a psychological background (mental health nurses and psychologists).

## 7 Conclusion

The analyses indicated that an original interest in definition, process and outcomes (White et al. 1998) was used to create a pool of items that did not have a good fit with Proctor's functions and that were not exclusively focused on effectiveness. The analysis of syntax revealed heterogeneity in MCSS‐26's wording and an uneven flow with negative/general questions being frontloaded. The CVI analyses suggest that only 12 of the 26 items were relevant for measuring effectiveness. We believe that quantitative clinical supervision research is budding and still in a context of discovery, and that a stronger operationalisation of relevant theoretical frameworks is needed before it can move into a context of justification.

## 8 Relevance to Clinical Mental Health Nursing Practice

If the MCSS‐26 is being used to assess the effectiveness of current clinical supervision practices within mental health organisations, researchers and evaluators must be mindful of its limitations and interpret MCSS scores with caution. Rather than making definitive claims about the effectiveness of supervision based solely on MCSS‐26 scores, researchers and evaluators should consider the broader context and, where possible, use additional measures to support their findings. This careful interpretation will help avoid overstating the effectiveness of current clinical supervision practices and lead to more meaningful clinical supervision‐related decision‐making within the mental health organisations.

## Author Contributions

All authors listed meet the authorship criteria according to the latest guidelines of the International Committee of Medical Journal Editors, and all authors are in agreement with the manuscript.

## Ethics Statement

The authors have nothing to report.

## Conflicts of Interest

The authors declare no conflicts of interest.

## Data Availability

The data that support the findings of this study are available from the corresponding author upon reasonable request.

## References

[inm70128-bib-0001] Australian College of Mental Health Nurses , Australian College of Midwives , and Australian College of Nursing . 2024. “Position Statement. Clinical Supervision for Nurses & Midwifes Australian College of Mental Health Nurses Australian College of Midwives Australian College of Nursing.”

[inm70128-bib-0002] Berry, S. , and N. Robertson . 2019. “Burnout Within Forensic Psychiatric Nursing: Its Relationship With Ward Environment and Effective Clinical Supervision?” Journal of Psychiatric and Mental Health Nursing 26, no. 7–8: 212–222. 10.1111/jpm.12538. 31209980

[inm70128-bib-0003] Butterworth, T. , J. Carson , E. White , J. Jeacock , A. Clements , and V. Bishop . 1997. It Is Good to Talk. School of Nursing, Midwifery, and Health VIsiting.

[inm70128-bib-0004] Carnie, A. 2021. Syntax: A Generative Introduction. 4th ed. Oxford University Press.

[inm70128-bib-0005] Edgar, D. , T. Moroney , and V. Wilson . 2024. “Enhancing New Graduate Nurses and Midwives Person‐Centredness Through Clinical Supervision During COVID‐19; Evaluation of a Non‐Randomized Intervention Study.” Journal of Advanced Nursing 80, no. 6: 2415–2428. 10.1111/jan.16012. 38097514

[inm70128-bib-0006] Gonge, H. , and N. Buus . 2015. “Is It Possible to Strengthen Psychiatric Nursing Staff's Clinical Supervision? RCT of a Meta‐Supervision Intervention.” Journal of Advanced Nursing 71, no. 4: 909–921. 10.1111/jan.12569. 25376433

[inm70128-bib-0007] Gonge, H. , and N. Buus . 2016. “Exploring Organizational Barriers to Strengthening Clinical Supervision of Psychiatric Nursing Staff: A Longitudinal Controlled Intervention Study.” Issues in Mental Health Nursing 37, no. 5: 332–343. 10.3109/01612840.2016.1154119. 27077399

[inm70128-bib-0008] Grant, J. S. , and L. L. Davis . 1997. “Selection and Use of Content Experts for Instrument Development.” Research in Nursing and Health 20, no. 3: 269–274. 10.1002/(sici)1098-240x(199706)20:3<269::aid-nur9>3.0.co;2-g. 9179180

[inm70128-bib-0009] Hamilton, J. , A. Cole , R. Bostwick , and I. Ngune . 2023. “Getting a Grip on Safewards: The Cross Impact of Clinical Supervision and Safewards Model on Clinical Practice.” International Journal of Mental Health Nursing 32, no. 3: 801–818. 10.1111/inm.13116. 36645077

[inm70128-bib-0010] Haynes, S. N. , D. C. S. Richard , and E. S. Kubany . 1995. “Content Validity in Psychological Assessment: A Functional Approach to Concepts and Methods.” Psychological Assessment 7, no. 3: 238–247.

[inm70128-bib-0011] Hyrkäs, K. , K. Appelqvist‐Schmidlechner , and L. Oksa . 2003. “Validating an Instrument for Clinical Supervision Using an Expert Panel.” International Journal of Nursing Studies 40, no. 6: 619–625. 10.1016/s0020-7489(03)00036-1. 12834927

[inm70128-bib-0012] Hyrkäs, K. , K. Appelqvist‐Schmidlechner , and M. Paunonen‐Ilmonen . 2003. “Translating and Validating the Finnish Version of the Manchester Clinical Supervision Scale.” Scandinavian Journal of Caring Sciences 17, no. 4: 358–364. 10.1046/j.0283-9318.2003.00236.x. 14629638

[inm70128-bib-0013] Khani, A. , M. Jaafarpour , and Y. Jamshidbeigi . 2009. “Translating and Validating the Iranian Version of the Manchester Clinical Supervision Scale.” Journal of Clinical and Diagnostic Research 3, no. 2: 1402–1407.

[inm70128-bib-0014] Landis, J. R. , and G. G. Koch . 1977. “The Measurement of Observer Agreement for Categorical Data.” Biometrics 33, no. 1: 159–174. 843571

[inm70128-bib-0015] Lynn, M. R. 1986. “Determination and Quantification of Content Validity.” Nursing Research 35, no. 6: 382–385. 3640358

[inm70128-bib-0016] Martin, P. , L. Lizarondo , S. Kumar , and D. Snowdon . 2021. “Impact of Clinical Supervision on Healthcare Organisational Outcomes: A Mixed Methods Systematic Review.” PLoS One 16, no. 11: e0260156. 10.1371/journal.pone.0260156. 34797897 PMC8604366

[inm70128-bib-0017] Masamha, R. , L. Alfred , R. Harris , S. Bassett , S. Burden , and A. Gilmore . 2022. “'Barriers to Overcoming the Barriers': A Scoping Review Exploring 30 Years of Clinical Supervision Literature.” Journal of Advanced Nursing 78, no. 9: 2678–2692. 10.1111/jan.15283. 35578563 PMC9546137

[inm70128-bib-0018] McCutcheon, K. , P. O'Halloran , and M. Lohan . 2018. “Online Learning Versus Blended Learning of Clinical Supervisee Skills With Pre‐Registration Nursing Students: A Randomised Controlled Trial.” International Journal of Nursing Studies 82: 30–39. 10.1016/j.ijnurstu.2018.02.005. 29574394

[inm70128-bib-0019] Oppenheim, A. N. 1992. Questionnaire Design, Interviewing and Attitude Measurement. 2nd ed. Continuum.

[inm70128-bib-0020] Polit, D. F. , and C. T. Beck . 2006. “The Content Validity Index: Are You Sure You Know What's Being Reported? Critique and Recommendations.” Research in Nursing and Health 29, no. 5: 489–497. 10.1002/nur.20147. 16977646

[inm70128-bib-0021] Proctor, B. 1987. “Supervision: A Co‐Operative Exercise in Accountability.” In Enabling & Ensuring, edited by M. Marken and M. Payne , 21–34. National Youth Bureau.

[inm70128-bib-0022] Rothwell, C.0. , A. Kehoe , S. F. Farook , and J. Illing . 2021. “Enablers and Barriers to Effective Clinical Supervision in the Workplace: A Rapid Evidence Review.” BMJ Open 11, no. 9: e052929. 10.1136/bmjopen-2021-052929. PMC8479981 34588261

[inm70128-bib-0023] Ryu, H. , N. Buus , L. Naccarella , H. Gonge , R. Prematunga , and B. Hamilton . 2024. “Characteristics of Clinical Supervision for Mental Health Nurses: A Survey Study Using the MCSS‐26.” Journal of Clinical Nursing 34: 2817–2829. 10.1111/jocn.17480. 39381879 PMC12181149

[inm70128-bib-0024] Schleef, E. 2013. “Written Surveys and Questionnaires.” In Research Methods in Sociolinguistics, edited by J. Holmes and K. Hazen . Wiley Blackwell.

[inm70128-bib-0025] Schwarz, N. , H.‐J. Hippler , and E. Noelle‐Neumann . 1991. “A Cognitive Model of Response‐Order Effects in Survey Meassurement.” In Context effects in social and psychological research. Springer‐Verlag, edited by N. Schwarz and S. Sudan . Springer‐Verlag.

[inm70128-bib-0026] Snowdon, D. , G. Millard , and N. F. Taylor . 2016. “Effectiveness of Clinical Supervision of Allied Health Professionals.” Journal of Allied Health 45, no. 2: 113–121. 27262469

[inm70128-bib-0027] Watkins, C. E. 2020. “What Do Clinical Supervision Research Reviews Tell Us? Surveying the Last 25 Years.” Counselling and Psychotherapy Research 20, no. 2: 190–208. 10.1002/capr.12287.

[inm70128-bib-0028] White, E. , T. Butterworth , V. Bishop , J. Carson , J. Jeacock , and A. Clements . 1998. “Clinical Supervision: Insider Reports of a Private World.” Journal of Advanced Nursing 28, no. 1: 185–192. 10.1046/j.1365-2648.1998.00743.x. 9687147

[inm70128-bib-0029] White, E. , and J. Winstanley . 2010. “A Randomised Controlled Trial of Clinical Supervision: Selected Findings From a Novel Australian Attempt to Establish the Evidence Base for Causal Relationships With Quality of Care and Patient Outcomes, as an Informed Contribution to Mental Health Nursing Practice Development.” Journal of Research in Nursing 15, no. 2: 151–167. 10.1177/1744987109357816.

[inm70128-bib-0030] Winstanley, J. 2000. “Manchester Clinical Supervision Scale.” Nursing Standard 14, no. 19: 31–32. 10.7748/ns.14.19.31.s54. 11209386

[inm70128-bib-0031] Winstanley, J. , and E. White . 2011. “The MCSS‐26: Revision of the Manchester Clinical Supervision Scale Using the Rasch Measurement Model.” Journal of Nursing Measurement 19, no. 3: 160–178. 10.1891/1061-3749.19.3.160. 22372092

[inm70128-bib-0032] Zonneveld, D. , T. Conroy , and L. Lines . 2025. “Clinical Supervision Models and Frameworks Used in Nursing: A Scoping Review.” Journal of Advanced Nursing 81, no. 7: 3583–3599. 10.1111/jan.16678. 39673208

